# Mental health and its relationship with social support in Iranian students during the COVID-19 pandemic

**DOI:** 10.1186/s40359-021-00589-4

**Published:** 2021-05-17

**Authors:** Reza Ghafari, Mojgan Mirghafourvand, Mahsa Rouhi, Shirin Osouli Tabrizi

**Affiliations:** 1 Medical Education Research Center, Health Management and Safety Promotion Research Institute, Tabriz University of Medical Sciences, Tabriz, Iran; 2 Department of Midwifery, Social Determinants of Health Research Center, Tabriz University of Medical Sciences, Tabriz, Iran; 3 Department of Midwifery, Students Research Committee, Faculty of Nursing and Midwifery, Tabriz University of Medical Sciences, Tabriz, Iran

**Keywords:** Mental health, Social support, Covid-19, Student

## Abstract

**Background:**

In addition to physical problems, the COVID-19 crisis continues to impose serious psychological adverse effects on people's mental health, which plays a major role in the efficiency of every community. Students, especially medical sciences students, suffer from more stress as a result of exposure to COVID-induced stressors. It is, therefore, essential to measure mental health and its relationship with social support in medical sciences students during the COVID pandemic. The present study was conducted to determine the mental health status of students and its correlation with social support.

**Methods:**

This cross-sectional study was conducted using random sampling on 280 students of Tabriz University of Medical Sciences in Iran in 2020. Socio-demographic profile scale, Mental Health Test (GHQ-28), and the scale of Perceived Social Support (PRQ-85) were used to collect data. Participants completed the questionnaires online.

**Results:**

Considering the potential confounding variables, a general linear model (GLM) was utilized to determine the relationship between mental health and perceived social support. Mean (± standard deviation) of total mental health score 26.5 (12.5) was in the acceptable range of 0–63., and 56% of students suffered from a mental disorder. Mean (± standard deviation) of social support score 128.2 (21.0) ranged from 25 to175. According to Pearson's correlation coefficient, there was a significant inverse correlation between social support score and total mental health score and all its subscales [*p* < 0.001; r =  − 0.294 to − 0.536]. According to the GLM, mental health score decreased significantly with social support score [*p* = 0.0001; − 0.32 to − 0.20; CI 95%; B = 0.26].

**Conclusions:**

Given the inverse relationship between social support and mental health, it is suggested to increase the level of social support for students at all times, especially during the stressful COVID-19 pandemic to improve their mental health.

## Background

In December 2019, cases of unusual coronavirus-induced pneumonia were reported in Wuhan, China, and the World Health Organization (WHO) declared the disease as a pandemic in 2020 [1]. The first cases of COVID-19 were publicly announced on Feb 19, 2020, and the first case of COVID-19 in Tabriz was reported on February 28, 2020 [2]. The pandemic and public health measures to decelerate the progression of the disease have caused profound changes in people's lifestyle and perception, which can have a serious impact on their mental health in addition to physical problems [3, 4]. These impacts include a dramatic increase in mental disorders, including anxiety, depression, stress, sleep disorders, and fear, among individuals [5]. Similarly, due to the closure of universities, restrictions on education, and the presence of medical students in the hospital, the COVID-19 pandemic has imposed substantial stress on students’ mental health [6]. According to the WHO, mental health is more than merely the absence of mental disorders. It includes mental well-being, perception of self-efficacy, independence and autonomy, adequacy and competence, intergenerational dependence, and self-fulfillment of potential intellectual and emotional abilities [7]. Inarguably, mental health plays a major role in the efficiency of any society [8].

Students as human resources have a special position and mental health has a significant impact on their academic and professional progress [9]. In addition, students of medicine experience considerable stress as a result of these factors due to being exposed to associated stressors caused by the fear of infection, loss of control and spread of the virus, feeling helpless due to failure to save patients, long working hours, and lack of protective equipment [10]. Factors associated with mental health include gender, socio-economic status, self-acceptance, religious beliefs, culture, and social support [11–14].

Social support is defined as the knowledge of the environment and a person's confidence in available help and support if needed. The results of studies showed significant relationships between social support and people's physical and mental status, life satisfaction, and quality of life; it is also known as a stress reliever [15–18]. According to Alipour et al. [19], perceived social support leads to increased mental health and social adjustment among students. In a study conducted in China, Cao et al. [20] realized that the prevalence of COVID-19 affected mental health and caused varying degrees of anxiety among students. They also found that their anxiety increased by economic factors, delay in starting university, and the impact of the pandemic on daily life. A negative correlation was also reported between social support and symptoms of anxiety during the pandemic [21, 22]. In a study conducted by Ye et al. [23], a positive correlation was shown between acute stress disorder, on the one hand, and stressful experiences and maladaptive coping strategies, on the other. However, a negative correlation was observed when providing appropriate social support and using adaptive techniques.

Li et al. [24] showed that the pandemic outbreak intensified disease-related consequences, such as anxiety and depression in students, and receiving more social support reduced the consequences of psychological symptoms, which developed sharply in the absence of providing proper social support. In a study conducted in Iran on students of medicine at Tehran University of Medical Sciences, no significant difference was reported for depression and anxiety before and during the pandemic, but the somatic symptoms of depression increased during the COVID pandemic [25]. The findings of these studies collectively indicate that special attention and strategies appropriate for students are essential to deal with mental issues resulting from the pandemic.

Given the psychological impacts [26] of the pandemic and the effect of mental disorders on students' academic performance [27], mental health promotion has become a major health policy agenda in universities. Social support is a possible strategy to promote mental health. The present study, therefore, was conducted to determine mental health status and its correlation with social support in Iranian students of medicine during the COVID pandemic.

## Methods

### Study design and procedures

This was a cross-sectional study conducted on students of medical sciences (Medicine, Dentistry, Pharmacy, Nursing-Midwifery, Health and Nutrition, Rehabilitation, Medical and Paramedical Management, and Information) in Tabriz University of Medical Sciences from September 22 to December 20, 2020. This study was approved by the ethics committee of the research and technology deputy of Tabriz University of Medical Sciences (IR.TBZMED.REC.1399.1158).

The inclusion criterion was being a student at Tabriz University of Medical Sciences and exclusion criteria were not providing an answer to over 20% of the questions, taking antidepressants or psychotic medication, or self-reported history of mental illness.

### Sampling

Proportional random sampling was conducted based on faculties. The list of senior students of each faculty (third-year students in medical, dentistry, and pharmacy fields and final-year students in other fields), along with their telephone numbers, was obtained from the Education Departments. To protect the information of the students, the education office of the university provided only the students' telephone numbers and their names were unknown to the authors. The number of students selected from each faculty was calculated according to the sample size of the study, and then participants were randomly selected from the list of students using the www.random.org website. The objectives and methodology of the study were then explained to the selected students. Consent forms and questionnaires were sent via WhatsApp to those who were willing to participate in the study. The consent forms were coded the same as the codes of the questionnaires. After filling the written consent forms, the participants completed the first part of the online questionnaire anonymously. The written consent forms containing information on the objectives of the study, confidentiality, and exclusion from the study in the case of dissatisfaction were provided to all the participants. Due to the restrictions imposed on other messengers in Iran, the use of WhatsApp by the majority of students, and the absence of students in the university, this messenger as an accepted and secure messenger in Iran was selected in this study*.*

### Measures

Socio-demographic profile scale, Mental Health Test (GHQ-28 = General Health Questionnaire-28), and the Scale of Perceived Social Support (PRQ-85-Part 2 = Personal Resource Questionnaire-85-Part 2) were used to collect data.

### Socio-demographic profile scale

The questionnaire included questions on age, parents’ education, major, employment status during studies, adequacy of family income for living expenses, place of residence, ethnicity, and high-risk behaviors, such as smoking cigarettes and hookahs, as well as alcohol abuse.

### GHQ-28

General health questionnaire-28 included four dimensions of physical symptoms, anxiety symptoms, social dysfunction, and depression symptoms, each containing 7 items. Scoring was performed using a four-point Likert scale: not at all = 0, slightly = 1, very = 2, and extremely = 3. Higher scores indicated lower mental health. The GHQ-28 incorporates four subscales, including somatic symptoms (items 1–7), anxiety and insomnia (items 8–14), social dysfunction (items 15–21), and severe depression (items 22–28). The questionnaire is concerned with the manifestation of new phenomena of a distressing nature within the last few weeks [28]. Scores over 21 for total score and over 7 for subscales demonstrated the presence of mental distress in students of the Tabriz nursing and midwifery school [29]. The validity and reliability of the questionnaire were confirmed in Iran by Taghavi [30] who reported a Cronbach's alpha coefficient of 0.90.

### PRQ-85-Part 2

The PRQ-85-Part 2 [31] measures the current state of social support, consisting of two parts. The second part, which was used in this study, measures perceived social support and includes five dimensions, namely friendship, assistance, social cohesion, value, and care. The questionnaire contains 25 items scored using a 7-point Likert scale from 1 to 7 (Strongly Disagree = 1, Disagree = 2, moderately Disagree = 3, Undecided = 4, Moderately Agree = 5, Agree = 6, and Strongly Agree = 7). It is worth mentioning that the validity and reliability of the questionnaire were previously confirmed for hemodialysis patients in Iran. The content validity and retest method were used to determine the validity and reliability of the questionnaire, respectively, with a correlation coefficient of 8.0 [32].

### Sample size

A sample size of 253 participants was determined using G*Power with a two-sided ɑ level of 0.05, a study power of 99%, and a correlation coefficient of 0.265 based on a study by Riahi et al. [33]. A final sample size of 280 individuals was determined given a 10% loss of samples.

### Statistical analysis

Data were analyzed in SPSS 24. Mean (± standard deviation) and frequency (percentage) were used to describe the socio-demographic characteristics. The normality of the quantitative data was determined and confirmed by skewness and kurtosis. The Pearson correlation test was used to determine the correlation between mental health and its components with perceived social support in the bivariate analysis. Independent t-test and one-way analysis of variance (ANOVA) were used to determine the relationship between socio-demographic characteristics and the total mental health score. Then, the variables related to the total mental health score (*p* < 0.05) were entered into the general linear model (GLM) as possible confounding variables along with the social support variable.

## Results

The mean (± SD) age of students was 22.8 (2.6) years. Less than half of the participants (43.6%) were male and the rest were female. Over one-third of the students (38.6%) were studying in the General Medicine program and over half of them (59.6%) were undergraduate students of medical sciences. The majority of participants (92.1%, 91.8%) reported that they did not smoke cigarettes or hookahs. About half of the participants (47%) were the first child in a family of four members. One-fifth of the students (21.4%) were employed. Nearly half of the students' mothers (45%) and about one-third of the student’s fathers (31%) had an academic degree. Two-thirds of the students (66.8%) had a normal body mass index (BMI), and less than one-fifth (15.4%) reported that their monthly incomes were inadequate for life expenses (Table 1).

**Table 1:** Socio-demographic characteristic of Iranian students and their relationship with mental health (n = 280)

Characteristics	N (%)*	Mental health Mean (SD)	*P*	Characteristics	N (%)*	Mental health Mean (SD)	*P*
*Age*			0.501^†^	*Mother’s education*			0.869^‡^
Lower than 22	160 (57.1)	26.0 (12.0)		Illiterate	16 (5.7)	25.9 (11.7)	
22 and more	120 (42.9)	27.1 (13.0)		Primary	31(11.1)	25.0 (12.6)	
*Gender*			0.401^†^	Secondary	33 (11.8)	28.3 (13.4)	
Male	122 (43.6)	26.5 (13.1)		High school	74 (26.4)	27.0 (12.9)	
Female	158 (56.4)	26.6 (12.1)		University	125 (45.0)	26.3 (12.2)	
*Educational grade*			0.749^†^	*Father’s education*			0.545^‡^
Bachelor	167(59.6)	26.7 (12.4)		Illiterate	5 (1.8)	35.2 (17.07)	
Doctorate	108(38.6)	26.5 (12.9)		Primary	25 (8.9)	23.8 (11.7)	
*Smoking*			0.003^†^	Secondary	24 (8.6)	26.0 (11.5)	
Yes	22 (7.9)	34.1 (15.4)		High school	3(1.1)	27.6 (7.6)	
No	258 (92.1)	25.9 (12.1)		Diploma	76 (27.1)	27.5 (11.7)	
*Hookah*			0.228^†^	University	147 (52.5)	26.3 (13.2)	
Yes	23 (8.2)	25.7 (11.1)		*Father’s occupation*			0.277^‡^
No	257 (91.8)	26.6 (12.7)		Unemployed	8 (2.9)	32.1 (16.8)	
*Smoking duration (Month)*			0.217^‡^	Worker	5 (1.8)	19.6 (8.1)	
< 12	14 (5)	29.4 (13.3)		Employee	72 (25.7)	25.05 (12.7)	
12–36	6 (2.1)	27.6 (13.1)		Physician	16 (507)	27.4 (11.5)	
> 36	7 (2.5)	39.5 (14)		Freelance	172 (61.4)	26.8 (12.3)	
*Number of family members*			0.343^‡^	*Number of child in family*			0.177^‡^
≤ 3	43 (15.4)	25.7 (13.2)		1	21 (7.5)	24.5 (12.9)	
4	146 (52.1)	27.8 (12.8)		2	151 (53.9)	27.9 (12.7)	
5	51 (18.2)	25.4 (12.9)		3	57 (20.4)	26.1 (13.0)	
≥ 6	40 (14.3)	24.2 (9.8)		≥ 4	51 (18.2)	23.8 (10.9)	
*Employed with education*			0.002^†^	*Birth order in family*			0.787^‡^
Yes	60 (21.4)	30.03 (13.1)		1	133 (47.5)	27.3 (13.3)	
No	220 (78.6)	25.6 (12.2)		2	87 (31.1)	25.8 (11.3)	
*Mothers occupation*			0.228^†^	3	29 (10.4)	25.4 (13.1)	
Employed	89 (31.8)	28.2 (13.6)		≥ 4	29 (10.4)	26.3 (12.6)	
Housewife	191(68.2)	25.8 (11.9)		*Adequacy of income for family expenses*			0.238^‡^
*BMI* (kg/m^2^)			0.003^‡^	Adequate	73 (26.1)	26.3 (12.5)	
Underweight (< 18.5)	17 (6.1)	24.9 (8.2)		Fairly adequate	167 (58.6)	25.9 (12.4)	
Normal (18.5–24.9)	187 (66.8)	25 (11.2)		Inadequate	43 (15.4)	26.6 (12.6)	
Overweight (25.0–29.9)	64 (22.9)	30.0 (15.5)					
Obese (≥ 30)	9(3.2)	36.3 (13.5)					

*Number (Percent)

^†^Independent t-test

^‡^One Way ANOVA

The mean (SD) of the total GHQ-28 score was 26.5 (12.5) in the obtainable range of 0–63. More than half of the students (56%) reported the presence of mental distress. The highest and the lowest mean scores were obtained for social performance [8.6 (2.5)] and depression, respectively [4.9 (4.5)]. The mean (SD) of the PRQ-85 score was 128.2 (21.0) within the range of 25–175. According to Pearson's correlation coefficient, there was a significant inverse correlation between social support score and total mental health score and all its subscales [r = ** − **0.294 to − 0.536; *p* < 0.001] (Table 2).

**Table 2:** The status of mental health and its domains and social support and their correlation among Iranian students (n = 280)

Variable	Mean (SD)	Obtained score range	Obtainable score range	Correlation with social support r (*p*)*
Total score of mental health	26.5 (12.5)	6–70	0–84	− 0.48 (< 0.001)
Physical health	6.1 (3.8)	0–21	0–21	− 0.29 (< 0.001)
Anxiety	6.8 (4.6)	0–21	0–21	− 0.32 (< 0.001)
Depression	4.9 (4.5)	0–21	0–21	− 0.54 (< 0.001)
Social function	8.6 (2.5)	4–20	0–21	− 0.37 (< 0.001)
Social support	128.2 (21.04)	60–168	25–175	–

*Pearson correlation test

According to the one-way ANOVA and independent t-test, there was a statistically significant relationship between mental health and the variables of smoking (*p* = 0.003), employment during studies (*p* = 0.002), and BMI (*p* = 0.003). These variables were entered into the GLM along with the social support variable. According to the GLM, mental health score decreased significantly with increasing the social support score [B = 0.26; CI 95% = ** − **0.32 to − 0.20; *p* < 0.001] (Table 3).

**Table 3:** Relationship between social support and metal health among Iranian students based on General Linear Model (n = 280)

Variable	Β (95% confidence interval)	*P* value
*Social support*	− 0.26 (− 0.32 to − 0.20)	< 0.001
*BMI* (kg/m^2^)		
Underweight (< 18.5)	− 5.07 (− 14.13 to 3.98)	0.271
Normal (18.5–24.9)	− 5.09 (− 12.63 to 2.45)	0.185
Overweight (25.0–29.9)	− 2.67 (− 10.39 to − 5.04)	0.496
Obese (≥ 30) (Reference)	0	
*Employee with education*		
Yes	1.63 (− 1.66 to 4.93)	0.329
No (Reference)	0	
*Smoking*		
Yes	4.90 (− 0.02 to 9.81)	0.051
No (Reference)	0	

## Discussion

In addition to physical health concerns, COVID-19 is associated with psychological disorders and affects the mental health of people in different communities [3]. Students' mental health is a major public health concern. It is also important to examine and prevent mental health disorders during the COVID-19 pandemic [34]. Given these conditions, the present study aimed to investigate mental health and its correlation with social support in Iranian students during the COVID-19 pandemic. The results of the present study showed that 56% of students reported the presence of symptoms of mental disorder and received only average social support. There was a significant relationship between social support and mental health.

In the present study, over half of the students reported the presence of mental disorder symptoms, which is higher than the pre-COVID-19 period given by studies conducted in this field. For instance, in a cross-sectional study carried out by Solhi et al. [35] before the COVID-19 pandemic on Iranian students of medicine using a researcher-made questionnaire on self-assessment of mental health, only 14.7% suffered from poor mental health. Furthermore, in a cross-sectional study conducted on Payam-e Noor students in West Azerbaijan province, Iran, 46.6% of students reported the presence of mental disorder symptoms based on the GHQ-28, which were more common among female students [36]. Results of a meta-analysis performed by Zare et al. [37] on studies that investigated the mental health of Iranian students using the GHQ-28 revealed that 32.3% of students reported the presence of symptoms of mental disorder. A previous study reported 38.1% mild to severe anxiety and 27.4% depression among students of medicine at Tehran University of Medical Sciences during the COVID-19 pandemic. The most common reported symptoms were changes in sleep patterns and anxiety. In addition, a higher level of anxiety was associated with the female gender, having COVID symptoms, and a low-grade point average [25]. Given the results of previous studies, the prevalence of mental distress has been on the rise among Iranian students during the COVID pandemic. However, all the above-mentioned studies used screening tools and were therefore unable to establish the prevalence of mental disorders.

There was no significant difference between the mental health of male and female students in the present study. In another study conducted during the COVID pandemic on 69,054 French students, severe symptoms of mental problems were reported in students who were under quarantine. According to this study, 11.4% of participants reported suicidal thoughts, 16.1% reported severe depression, 27.5% suffered from high levels of anxiety, and only 12.45% visited a doctor. The study indicated that health issues during the COVID pandemic were more prevalent among French students [34]. In a study on 476 Bangladeshi students during the COVID pandemic, the authors shared standard questionnaires with students through social media and found that 15% of the students showed symptoms of medium depression and 18.1% suffered from severe depression. In addition, depression was more common among students who studied in tuition-paying universities. Financial problems were reported to be a cause of depression and anxiety among students [5].

The results of the present study indicated a significant moderate inverse correlation between mental health and social support in Iranian students. A significant statistical correlation between social support and mental health was reported in other studies in the pre-COVID period [38, 39]. Results of a study on 450 Chinese students of various majors during the COVID pandemic indicated a positive correlation between COVID-induced stressors and psychological symptoms, such as depression and anxiety, and there was a negative correlation between social support and psychological symptoms [24]. Results of another study conducted on 2020 individuals during the COVID pandemic indicated that those who experienced isolation and loneliness reported higher rates of depression. In addition, low social support was significantly associated with an elevated risk of depression and poor sleep quality [40]. Given the importance of social support and the COVID-induced mental health crisis, conditions must be created for students so that they feel there are people who can support them in difficult situations. Special plans should also be developed to improve mental health and identify students to refer them to counseling centers [13, 27, 41].

Given that the COVID-19 has brought unprecedented disruption to social life, universities must therefore provide social support, such as providing counseling through social media, due to these conditions. To promote mental health in students, organizations and institutions should also provide their access to mental health services using both face-to-face and digital platforms. Telemental health services should be provided for students [42]. For this purpose, the WHO and UNICEF are holding a series of webinars, entitled "coping with COVID", which mainly aim to establish a real relationship with young people and encourage them to ask their questions from the experts of the WHO and UNICEF to increase mental health awareness among young people and strengthen the demand for psychological interventions [43, 44].

The strengths of the present study were random sampling and including students from all faculties of Tabriz University of Medical Sciences, both of which strengthened the generalizability of the results. The cross-sectional nature of the study was one of its limitations, and the correlations shown here failed to accurately indicate a causal relationship. Correlation analyses do not allow establishing a causal relationship. A relationship of cause-effect should be derived from elements related to the design of a study (follow-up data, dose–effect relationship, control of confounding variables, etc.). Lack of pre-pandemic data within the same study population and low sample size were the other limitations of this study. Since this study was performed on healthy students, the results cannot be generalized to people who have a history of taking antidepressants or depression. Therefore, conducting similar studies is recommended on different societies with different cultures in future research.

## Conclusions

Our results indicated that more than half of students might suffer from a mental disorder, which highlights the importance of screening and designing appropriate interventions in this regard. Considering the inverse correlation between social support and mental health in this study, it is suggested to promote students' mental health by providing them with higher levels of social support, especially during the stressful COVID-19 pandemic.

## Data Availability

The datasets and analyzed in this study can be made available by the corresponding author at reasonable request.

## Abbreviations

WHO: World Health Organization
Covid-19: Coronavirus disease
PRQ-85-Part 2: Personal Resource Questionnaire-85-Part 2
GHQ-28: General Health Questionnaire-28
